# Innocuity and immune response to *Brucella melitensis* Rev.1 vaccine in camels (*Camelus dromedarius*)

**Published:** 2014-10-25

**Authors:** A. Benkirane, A.H. El Idrissi, A. Doumbia, K. de Balogh

**Affiliations:** 1 *Department of Pathology and Veterinary Public Health, Institut Agronomique et Vétérinaire Hassan II, Rabat, Morocco*; 2 *Animal Production and Health Division, Food and Agriculture Organization of the United Nations, Rome, Italy*

**Keywords:** *Brucella melitensis*, Camel, Milk, Rev.1 vaccine

## Abstract

A field trial was conducted in a camel brucellosis-free herd to evaluate antibody response to the *Brucella melitensis* Rev.1 vaccine in camels and assess shedding of the vaccine strain in milk. Twenty eight camels were divided into four groups according to their age and vaccination route. Groups A (n=3) and B (n=3) consisted of non-pregnant lactating female camels, vaccinated through subcutaneous and conjunctival routes, respectively. Groups C (n=10) consisted of 8-11 months old calves vaccinated through conjunctival route. The rest of the herd (n=12) composed of female and young camels were not vaccinated and were considered as the control group. Each animal from groups A, B and C was given the recommended dose of 2 × 10^9^ colony forming units of Rev.1 vaccine irrespective of age or route of vaccination. Blood samples were collected from all the animals at the time of vaccination and at weekly, bi-weekly and monthly interval until 32 weeks post vaccination and from controls at weeks 8 and 24. The serological tests used were modified Rose Bengal Test, sero-agglutination test, and an indirect Enzyme Linked Immunosorbent Assay. Milk samples were collected from all vaccinated female camels and tested for the presence of Rev.1 vaccine strain. Most vaccinated animals started to show an antibody response at week 2 and remained positive until week 16. By week 20 post-vaccination all animals in the three groups were tested negative for *Brucella* antibodies. Bacteriological analysis of milk samples did not allow any isolation of *Brucella melitensis*. All samples were found *Brucella* negative in PCR analysis. The results of this study indicate that the Rev.1 vaccine induces seroconversion in camels. Rev.1 vaccine strain is not excreted in the milk of camels. These findings are promising as to the safe use of the Rev.1 vaccine in camels.

## Introduction

Dromedary (*Camelus dromedarius*) farming is important throughout countries of North Africa, the Horn of Africa and the Middle East, not only as a transport means for nomads but also as leather, wool, milk and meat provider for local consumption, as well as an important sport and tourism resource in the Arabian Gulf countries. Despite the growing importance of camel farming, the epidemiological features and investigations of infectious disease problems have not been the focus of many studies in this animal species (Tibary *et al.*, 2006).

In particular, brucellosis in camels has not received much attention from researchers and scientists (Abbas and Agab, 2002; Gwida *et al.*, 2012). Camelids are not known to be primary nor main hosts of *Brucella spp*., but they are susceptible to both *B. abortus* and *Brucella melitensis* (*B. melitensis*) (Cooper, 1991; Abbas and Agab, 2002; Gwida *et al.*, 2012). Yet, in addition to causing abortions, stillbirths and other clinical signs in camels, brucellosis is a zoonotic disease which can spread and cause disease to humans especially those in contact with infected animals and those consuming milk or dairy products usually manufactured using traditional methods (Cooper, 1991; Benkirane, 2006).

*Brucella* infection rate and the contribution of infecting *Brucella* species in a given country are correlated with the prevalence of brucellosis in the primary animal host species i.e: cattle, sheep, and goats, respectively for *B. abortus* and *B. melitensis*. However, in the last two decades, there has been an apparent increase in the prevalence of brucellosis in small ruminants in many countries in Central Asia and Eastern Europe because of various sanitary and socioeconomic reasons including the breakdown of disease control systems in many former soviet republics (Jackson *et al.*, 2007; Ward *et al.*, 2012). The situation of brucellosis in Middle East is also worsening (Pappas *et al.*, 2006) presumably because of lack of strict disease control and surveillance programmes. *B. melitensis* emerged as a causative agent of bovine and cameline brucellosis, especially in some Middle Eastern countries (Benkirane, 2006) when they are pastured together with infected sheep and goats. Milk from infected camels represents a major source of infection that is underestimated in the Middle East (Musa *et al.*, 2008). *B. melitensis* biovar 3 is the most widespread source of infection in camels in the Middle East, and it has been isolated in Sudan, Jordan and Egypt. *B. melitensis* biovar 1 has also been isolated in Iran, Kuwait and Libya. The reported prevalence varied between a low prevalence (2-5%) in nomadic or extensively kept camels to a high prevalence (8-15%) in camels kept intensively or semi-intensively (Abbas and Agab, 2002).

The *B. melitensis* Rev.1 vaccine (Rev.1 vaccine) is the best vaccine available for the control of brucellosis in small ruminants (Blasco, 1997, 2006; Munoz *et al.*, 2008). In camels, although vaccination with the *B. melitensis* Rev.1 strain has been occasionally applied (Radwan *et al.*, 1995), its innocuity and protective efficacy have been poorly documented. With the emergence of *B. melitensis* in camels, it is expected that affected countries with a big camel industry will use the Rev.1 vaccine to protect their herds against this infection. The Rev.1 vaccine is infectious to humans and its use in lactating females including camels could be a hazard for consumers through consumption of unpasteurized milk. A limited number of confirmed cases have been reported as being of sheep and goat origin (Blasco and Diaz, 1993; Banai *et al.*, 1995; Bardenstein *et al.*, 2002) and others of camel origin (Ben Shimol *et al.*, 2012; Gwida *et al.*, 2012).

The present study was conducted to evaluate, in a field trial, the innocuity and immune response to Rev.1 vaccine in camels and assess the bacterial shedding of the Rev.1 vaccine strain in the milk of female camels.

## Materials and Methods

### Animals

Twenty-eight local Guerzini “type” camels included in this field study were obtained from a “brucellosis-free” state owned farm located in the region of Laayoune, south Morocco during the period between January 2012 and July 2012. Study animals were either females aged 5-11 years or calves aged between 8-11 months. Brucellosis was never reported and there is no history of brucellosis vaccination in the Laayoune region. Before the start of the trial, all animals were subjected to a thorough clinical examination. Milk samples from the female camels and blood samples from all animals were taken for testing before the experiment.

### Treatment groups and vaccination protocols

The *B. melitensis* Rev.1 vaccine (ND Ocurev; CZV Porriño. Spain) ready for conjunctival delivery was used in this experimental study. The dose administered via the conjunctiva was two drops (50 to 60 microliters) per animal in the same eye. For the subcutaneous route, the vaccine vial content was diluted in 40 ml sterile phosphate buffer saline (pH=7.4) and a dose of 2 ml inoculated to each animal at the elbow. The colony forming units (CFU) counts and the assessment of the absence of contamination and Rev.1 vaccine dissociation were performed on Trypticase Soy Agar before and after vaccination following standard procedures (Alton *et al.*, 1988).

Animals were assigned to three groups based on age, sex and lactation status. Groups A (n=3) and B (n=3) consisted of non-pregnant lactating female camels, vaccinated with Rev.1 vaccine through conjunctival and subcutaneous routes, respectively. Groups C (n=10) consisted of 8-11 months old calves similarly vaccinated through conjunctival (C) route. The rest of the animals in the herd, which consisted of 12 adult dry female camels, were not vaccinated and therefore considered as control group. The selection of study animals and design of treatment groups were decided according to the availability of animals rather than random selection.

After vaccination, all animals were reared together with no restriction of movements between vaccinated and unvaccinated animals. Animals were observed daily during the first 15 days post-vaccination for any adverse reactions.

### Serological testing

Blood samples from all vaccinated animals were collected before vaccination, and subsequently on a weekly basis for the first 8 weeks, biweekly from week 8 to 16 and every 4 weeks from week 16 to 32. Blood was also collected from control animals on weeks 8 and 24 in order to detect any possible horizontal passage of the vaccine strain bacteria between vaccinated and unvaccinated animals. All samples were centrifuged locally and refrigerated until ready for transport to the laboratory.

Collected sera were evaluated for antibodies to the Rev.1 vaccine strain by three serological methods, the modified Rose Bengal test (mRBT), a commercial sero-agglutination test (SAT), and a customized indirect ELISA (iELISA). Serum samples taken from control animals were also used for the design and standardization of the iELISA test.

A commercial Rose Bengal antigen (Synbiotics) was used in a modified test with 25µL antigen and 75µL serum as described by Blasco *et al*. (1994). Results were considered positive for RB when there was any degree of visible agglutination.

A commercially available sero-agglutination test (SAT) antigen (Synbiotics) was used to test the samples according to the manufacturer’s instructions. The SAT antigen was diluted ten-fold in phenicated physiological water (0.5%) and distributed in clean serology tubes together with test sera at dilutions ranging from 1/10 to 1/320 and a constant volume of 0.5 ml for both reactants. Due to the lack of a gold standard positive camel serum at the WHO/FAO/OIE reference laboratory for brucellosis, the Veterinary Laboratories Agencies, Weybridge, agglutinations at the dilutions of 1/20 and beyond were considered as positive.

An iELISA was developed and standardized as follows: The antigen (*B. melitensis* 16M S-LPS obtained by phenol extraction) was used at 2.5 µg/ml. Sera were diluted from 1/5 to 1/200. The highest differences between the optical density (OD) readings before vaccination and of the unvaccinated groups (considered as gold standard negative population) and three weeks after vaccination (maximal response, and considered as the gold standard positive population) was evidenced using the 1/5 serum dilution. As conjugates, both recombinant protein G and A/G (from Pierce) were tested at concentrations ranging 2-3 µg/ml. The best resolution using the same gold standard sera than above was obtained with the protein A/G at 3 µg/ml. The substrate was ABTS and the OD was assessed at 15, 20, 25 and 30 minutes at 405 nm. Antigen solution in Phosphate buffer solution (PBS) (2.5 µg/ml) was adsorbed to plastic plates (100 µl/well) after overnight incubation at 4ºC. Duplicate serum dilutions (1/5) were incubated (100 µl/well) at 37ºC for 45 min. The working dilution (100 µl/well of protein A/G at 3 µg/ml in PBS-Tween) of the conjugate was then incubated at 37ºC for 45 min, and the reaction revealed with 100 µl/well of ABTS substrate with readings (405 nm) at 15, 20, 25 and 30 min. The mean OD was expressed as the percentage OD of a control serum. This test was performed only on sera collected from week 0 to week 16 at the Centro de Investigación y Tecnología Agroalimentaria, Saragossa Spain (CITA).

### Testing of milk samples

Milk samples were collected twice weekly during the first eight weeks after vaccination. Milk samples were drawn into sterile tubes from the four teats of the mammary gland. Milk samples from each animal were pooled and stored at 4°C, and transported to the laboratory for immediate culture within a maximum of three days after sampling. The creamy layer and deposit from each sample were collected and spread onto the Farrell selective medium containing a commercial antibiotic supplement (Oxoid Ref SR0209E) and the CITA medium used according to De Miguel *et al*. (2011). The plates were incubated during 10 days at 37°C and regularly observed for any growth.

### DNA extraction and PCR

The Polymerase Chain Reaction (PCR) was performed as described by Mayer-Scholl *et al*. (2010) at the Brucellosis Unit of the Veterinary Laboratories Agencies, Weybridge. Briefly, DNA samples extracted from milk samples from each animal and also from the samples from which suspect colonies grew were tested by a multiplex PCR for the detection of *Brucella* species, including *B. melitensis* and the Rev.1 vaccine strain.

## Results

There were no clinical signs attributable to vaccine administration observed in any of the vaccinated animals. Slight temperature rises (ca. 1°C) occurred at day 7 post-vaccination in most animals, which look healthy for the rest of the field trial.

### Serological testing

All sera taken from control animals tested negative for antibodies to the Rev. 1 vaccine for all three serological tests, confirming the absence of *Brucella spp*. in the study location. Post-vaccination antibodies using mRBT are shown in Table 1. All animals of the three groups were antibody positive from week 2 to week 10 after which sero-positivity began to decline. At week 20 and beyond, all sera became negative.

**Table 1:** Number of seropositive camels in the treatments groups (A, B and C) by the modified Rose Bengal Test following vaccination with the *B. melitensis* Rev. 1 vaccine.

week	Group A (n=3)	Group B (n=3)	Group C (n=10)	Total positive
0	0	0	0	0
1	2	0	0	2
2	3	3	10	16
3	3	3	10	16
4	3	3	10	16
5	3	3	10	16
6	3	3	10	16
7	3	3	10	16
8	3	3	10	16
9	-	-	-	-
10	3	3	9	15
11	-	-	-	-
12	3	3	6	12
13	-	-	-	-
14	3	3	4	10
15	-	-	-	-
16	3	3	3	9
20	0	0	0	0

*Group A = 3 female camels vaccinated conjunctivally. Group B = 3 female camels vaccinated subcutaneously. Group C = 10 camel calves vaccinated conjunctivally.*

**Fig. 1:**
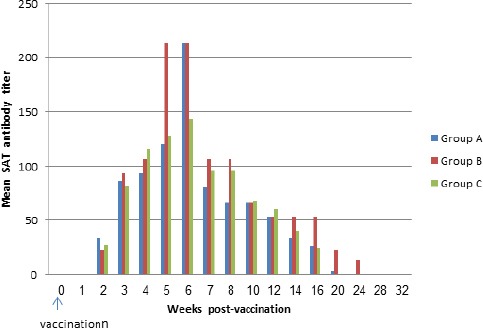
Mean antibody titers by Serum Agglutination Test in the treatment groups (A, B and C) following vaccination with *B. melitensis* Rev. 1 vaccine. *Group A = 3 female camels vaccinated conjunctivally. Group B = 3 female camels vaccinated subcutaneously. Group C = 10 camel calves vaccinated conjunctivally.*

Post-vaccination seroconversion (with a threshold of ≥1/20) was detected using the SAT in four animals from week 2, then in all animals from week 3 onwards. Detection of antibodies began to decline from week 16 until weeks 20 and 24 when only one animal from group B remained positive. Most animals showed high titers 5 or 6 weeks post-vaccination in the three treatment groups (Figure 1).

**Fig. 2:**
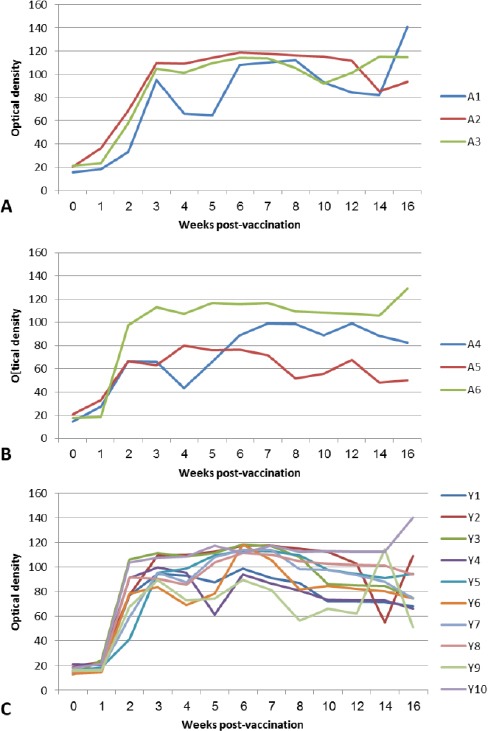
Antibody titers by iELISA in the treatment groups (A, B and C) following vaccination with the *B. melitensis* Rev. 1 vaccine. **(A):** Antibody response in Group A (3 she-camels, vaccinated cunjunctivally). **(B):** Antibody response in group B (3 she-camels, vaccinated subcutaneously). **(C):** Antibody response in group C (10 young calves, vaccinated cunjunctivally).

Figure (2) provides the temporal evolution of sero- conversion detected using the iELISA test for each treatment group. Most vaccinated animals in the three groups started to show an antibody response at week 2 which remained at high levels until week 16. Samples were not tested beyond week 16.

The seroconversion as measured by the three tests was similar with slight delay in terms of persistence of antibodies tested by SAT in group B where animals were vaccinated subcutaneously.

### Analysis of milk samples

All milk samples were culture negative on both culture media (Farrell’s and CITA) and found to be negative in PCR assay for *B. melitensis* and Rev.1 vaccine strain. The use of two distinct culture media is justified by the fact that nalidixic acid and bacitracin contained in the Farrell’s have some inhibitory effects on the growth of *Brucella*, particularly *B. melitensis*; therefore, other culture media such as the CITA medium should be used simultaneously with the Farrell’s to increase the likelihood of isolating smooth *Brucella* colonies (De Miguel *et al.*, 2011).

## Discussion

The administration of the Rev.1 vaccine in adult and young camels has not revealed any significant adverse reaction in vaccinated animals. This confirms observations from the field reports in Oman (El Idrissi, personal communication) where Rev.1 vaccine has been safely used in camels.

According to the manufacturer, the vaccine used in this trial meets the standards especially with regard to the possible smooth-rough dissociation that might lead to vaccine inefficacy. It is worth noticing that, in view of the smooth-rough dissociation drawback, it was recently suggested that some genetic modifications may stabilize the Rev.1 strain (Mancilla *et al.*, 2013).

Antibody response to vaccination as measured by mRBT, SAT and iELISA showed a standard seroconversion comparable to the serological evolution reported in other animal species such as cattle, sheep and goats. Although none of these tests has been evaluated in camels, they have been widely used to assess the serological response to *Brucella* infection in camels (Abbas and Agab, 2002). The modified Rose Bengal test (Blasco *et al.*, 1994) and an iELISA were found to be more sensitive than the conventional Rose Bengal and CFT when used to test animals for *B. melitensis* infection (Ferreira *et al.*, 2003). This justified the use of these two tests in addition to SAT in order to evaluate the serological response to Rev.1 vaccine in camels.

Given the late reproduction maturity and mating in female camels (ca. three years of age), on the one hand, and the duration of post-vaccination seropositivity not exceeding six months whatever route of vaccination was used and irrespective of age, on the other hand, one does not have to be strict concerning the age at which vaccination should be administered. Thus, should the above finding be corroborated through more extensive studies, 12 to 18 months could be the age at which vaccination is to be performed.

The number of animals in each subset is too low and does not allow for any statistical interpretation. However, it appears that adults react more profoundly than young animals, which is in line with reported findings in sheep and goats (Fensterbank *et al.*, 1982; Blasco, 2006). It is noteworthy that, on week 20, all sera became negative with mRBT and, on week 24, they were all negative when tested with SAT.

The role of the route of vaccination in the persistence of *Brucella* antibodies could not be assessed given the small number of animals in each group. It is known that, in small ruminants, antibodies persist much longer when animals are vaccinated subcutaneously than conjunctively (Zundel *et al.*, 1992; Verger, 1995).

The subcutaneous route of Rev.1 vaccine administration remains widely used in most countries, sometimes at so-called “reduced doses”, although it was demonstrated that the conjunctival route is preferable both in terms of safety (reduction of post-vaccination abortions in emergency situations when pregnant animals are vaccinated) and with respect to *Brucella* excretion in the milk (Blasco, 1997). This was demonstrated in small ruminants but never in camels and inference is made in this work assuming that camels will react alike small ruminants. However, it has not been proven that reducing the number of CFU per vaccine dose would preserve its full potency or confer it a better safety (Blasco, 1997). Thus, only the conventional dose of 1 to 2×10^9^ CFU was used in this work.

The absence of shedding of the vaccine strain in milk as tested by the lack of bacterial isolation up to 8 weeks was confirmed by PCR that failed to detect any trace of *Brucella* DNA in tested samples. Shedding of the Rev.1 vaccine strain through the udder following vaccination has occasionally been reported in sheep and goats. When used in pregnant ewes, the Rev.1 strain may lead to abortion and the excretion of the bacterium in the milk. In a field experiment, a few goats excreted Rev.1 strain in milk for 44 and 49 weeks post-abortion and in one ewe out of 19 (ca. 5 per cent) the excretion persisted for 6 months post-abortion (Zundel *et al.*, 1992). However, when vaccination was performed in non-pregnant goats, no vaccine strain was isolated in the milk (Jones and Marly, 1975). Similar results have been obtained in cows vaccinated with a reduced dose of Rev.1 vaccine (Garcia-Carrillo, 1980).

The only available study on the control of camel brucellosis was conducted in Saudi Arabia (Radwan *et al.*, 1995). No *Brucella* organisms were recovered in the Farrell’s medium from repeated udder secretion samples from all vaccinated milking camels. This finding is in line with our results though one should consider a higher number of vaccinated animals to confirm the absence of *Brucella* excretion in the milk.

## Conclusion

The present work showed that, when female camels were vaccinated against brucellosis with the Rev.1 vaccine administered either subcutaneously or conjunctively, this did not result in the shedding of the vaccine strain in the milk throughout a follow-up period of up to eight weeks. However, these results were obtained with only a small number of animals that is not significantly representative. Should this finding be confirmed through a study with more animals, it would be concluded that the milk from vaccinated animals does not yield *Brucella*. This would be a good argument in favor of vaccinating adult camels in case it is required, given the milk consumption habit of camel keepers and their families drinking raw milk from the udder. It is also recommended to verify these finding using a larger number of animals to refine the estimation of the duration of the post-vaccinal seroconversion. Finally, the most critical future step to be undertaken is to evaluate the vaccine safety in pregnant female camels as well as the potency through a vaccination-challenge trial conducted on a sufficient number of animals.
